# Advanced convolutional neural network modeling for fuel cell system optimization and efficiency in methane, methanol, and diesel reforming

**DOI:** 10.7717/peerj-cs.2113

**Published:** 2024-05-31

**Authors:** Sercan Yalcin, Muhammed Yildirim, Bilal Alatas

**Affiliations:** 1 Computer Engineering, Adiyaman University, Adiyaman, Turkey; 2 Computer Engineering, Malatya Turgut Ozal University, Malatya, Turkey; 3 Software Engineering, Firat (Euphrates) University, Elazig, Turkey

**Keywords:** Artificial intelligence, Convolutional neural networks, Fuel cell, Fuel reforming

## Abstract

Fuel cell systems (FCSs) have been widely used for niche applications in the market. Furthermore, the research community has worked on using FCSs for different sectors, such as transportation, stationary power generation, marine and maritime, aerospace, military and defense, telecommunications, and material handling. The reformation of various fuels, such as methanol, methane, and diesel can be utilized to generate hydrogen for FCSs. This study introduces an advanced convolutional neural network (CNN) model designed to accurately forecast hydrogen yield and carbon monoxide volume percentages during the reformation processes of methane, methanol, and diesel. Moreover, the CNN model has been tailored to accurately estimate methane conversion rates in methane reforming processes. The proposed CNN models are created by combining the 3D-CNN and 2D-CNN models. The Keras Tuner approach in Python is employed in this study to find the ideal values for different hyperparameters such as batch size, learning rate, time steps, and optimization method selection. The accuracy of the proposed CNN model is evaluated by using the root mean square error (RMSE), mean absolute percentage error (MAE), mean absolute error (MAE), and R2. The results indicate that the proposed CNN model is better than other artificial intelligence (AI) techniques and standard CNN for performance estimation of reforming processes of methane, diesel, and methanol. The results also show that the suggested CNN model can be used to accurately estimate critical output parameters for reforming various fuels. The proposed method performs better in CO prediction than the support vector machine (SVM), with an R2 of 0.9989 against 0.9827. This novel methodology not only improves performance estimation for reforming processes but also provides a valuable tool for accurately estimating output parameters across various fuel types.

## Introduction

The usage of hydrogen for the decarbonization of various sectors is one of the most discussed topics nowadays by the research community, governments and different organizations. Many reports and articles have been published to suggest a roadmap for hydrogen usage to decarbonize energy systems (Seck et al., 2022). However, there are several significant challenges to the wide implementation of hydrogen in different sectors such as transportation. Two of the most important challenges can be listed as hydrogen storage and distribution. To overcome these challenges, alternative fuels such as methanol, diesel and methane can be used as hydrogen carriers. These fuels can be converted to hydrogen by employing reforming technologies. It is also possible to produce alternative fuels *via* carbon dioxide hydrogenation by using hydrogen and carbon dioxide (Yue et al., 2021). Therefore, many research groups have discussed different aspects of fuel reforming.

Men et al. (2008) experimentally studied the performance of a methanol-reformed fuel system for low-power applications. The researchers used microstructured reactors in the system to convert methanol to syngas, and for other operations such as carbon monoxide removal. Kolb et al. (2012) also investigated the performance of a methanol steam-reformed high-temperature proton-exchange membrane (PEM) fuel cell (HT-PEMFC) system for net 5 kW power generation. The researchers in Kappis, Papavasiliou & Avgouropoulos (2021) tested a novel Pt/In_2_O_3_ catalyst for steam reforming of methanol. It should be noted that the researchers have been specifically interested in methanol-reformed fuel to feed the HT-PEMFC (Samsun et al., 2014) because HT-PEMFC can tolerate the CO in the methanol steam reformate gas and the operation temperatures of methanol steam reformer and HT-PEMFC are close. In addition, other unique features of methanol make it a suitable candidate for HT-PEMFC systems. However, other fuels can be also converted to syngas to feed the HT-PEMFC systems. For example, Samsun et al. (2014) conceptually designed and tested diesel and kerosene autothermal reformer systems to produce 5 kW electricity by employing an HT-PEMFC stack (Hardman, Chandan & Steinberger-Wilckens, 2015).

The suggested system in Samsun et al. (2021) is specifically suitable for auxiliary power applications in various sectors when long-run time and operation in extreme conditions are critical for the customers. Samsun et al. (2014) also investigated autothermal diesel reformate gas-fueled HT-PEMFC for auxiliary power applications. Their experimental work shows that diesel-based HT-PEMFC can have a high potential to be used for real applications on a commercial scale. The researchers have also widely investigated hydrogen production and different applications of methane reforming. Indeed, methane reforming has been commonly used for commercial-scale hydrogen production (Samsun et al., 2021). The effectiveness of methane reforming systems for solid oxide fuel cells (SOFCs) (Fan et al., 2022) and molten carbonate fuel cells has been the subject of numerous investigations. The hydrogen and carbon monoxide contents in the reformatted gas are critical output parameters for fuel reforming. Many studies have been performed to understand the change of hydrogen and carbon monoxide for reforming methanol, methane, and diesel with different catalysts (Consonni et al., 2021). In addition, many research groups have performed thermodynamic analyses to understand important operation parameters for reforming methanol, methane, and diesel.

Wang et al. (2022) worked on a novel Ni/CeO_2_ catalyst dry reforming of methane. Almithn & Alhulaybi (2022) performed a study about methanol steam reforming over Ni_2_P catalyst to understand pathways of the water-gas shift reaction, methanol decomposition, and methanol steam reforming over this catalyst. Chen et al. (2019) conducted experimental and simulation work to investigate the effects of operation parameters on H_2_ and CO yield in the diesel reforming process over Pt/CeO_2_-Al_2_O_3_ catalyst. Ju et al. (2019) analyzed the performance of enhanced Ni-Al-Based catalysts *via* electron microscopy for two types of diesel. The effects of the catalyst and the reaction kinetics are not taken into account in the thermodynamic study of fuel reforming. Thermodynamic analysis of fuel reformation is a useful technique for determining and comprehending the impact of key operational factors on fuel composition. Song, Han & Wang (2020) estimated syngas composition of the syngas obtained from steam reforming, partial oxidation, and autothermal reforming of petrol, diesel, and kerosene by employing a thermodynamic analysis.

Carapellucci & Giordano (2020) estimated hydrogen production from methane reforming by using thermodynamic analysis. Their thermodynamic analysis was based on Gibbs free energy minimization method and various reforming methods for methane were considered in the study. Recent trends in the literature are using machine learning (ML) methods to estimate the syngas composition of reformed fuels. Park et al. (2020) developed an ML model based on an artificial neural network (ANN) to estimate the performance of diesel reforming under different operation parameters for diesel engines. Nkulikiyinka et al. (2020) employed two ML methods, which are ANN and random forest (RF) models, to predict gas composition in sorption-enhanced steam methane reforming. They developed both ANN and RF models as soft sensor prediction models. The authors used the sorbent-to-carbon ratio, steam-to-carbon ratio, pressure, and temperature as input parameters to build the prediction models. Lee et al. (2021) implemented an ANN based on an actual operation dataset to estimate the syngas composition of methane reforming and optimize the steam methanol reforming (SMR) process. Yu et al. (2021) optimized a pressure swing adsorption process for high-purity hydrogen production from the SMR. The authors develop an ANN-based model with a Non-dominated sorting genetic algorithm-II (NSGA-II) to optimize the process. Byun et al. (2021) estimated the performance of SMR for hydrogen production by using ML-data-driven prediction models. The authors performed energy, environmental and techno-economic assessments of the SMR system by using Aspen Plus process simulation and MATLAB. They selected and compared Gaussian process regression (GPR), support vector regression (SVR), and decision tree (DT) regression to build the data-driven estimation model. The prediction models in Byun et al. (2021) were developed by using the MATLAB toolbox. Deng & Guo (2022) investigated the performance of bireforming of methane (BRM) by using ANN. They used reforming temperature, inlet carbon dioxide and steam flows to the reformer as input parameters to develop the ANN model. Then, the authors used the ML model to predict the syngas composition and rate of methane conversion.

It can be understood from the literature review mentioned above that the estimation of syngas composition of the reforming processes is a critical task. Specifically, the accurate estimation of hydrogen and carbon monoxide contents in the syngas is significant to coupled reforming processes with fuel cell systems. ML methods are promising tools to accurately estimate hydrogen and carbon monoxide in syngas. In the literature, the research community has generally used ANN to develop prediction models for reforming processes. The main goal of this article is to develop a general framework for accurate estimation of hydrogen and carbon monoxide content in the syngas produced from methanol, methane, and diesel. The researchers can use the proposed framework for performance estimation of the reformatted gas fueled fuel cell systems.

The other contributions and novelties of this study are listed as follows:

• In this study, unlike other studies in the literature, a powerful convolutional neural network (CNN) model is proposed to predict hydrogen yield, and CO ratio in the syngas for reforming methane, methanol, and diesel. Additionally, the CNN model has been customized to precisely gauge methane conversion rates during methane reforming procedures.

• The findings have been impacted by the preprocessing methods used on the data before feeding it into the CNN model. The capacity of the suggested model to learn from and generalize from the data is improved by using appropriate data preparation techniques, such as feature scaling and normalization.

• The proposed CNN model comprises both 2D and 3D convolution, max pooling, feature map convolution, and other layers that will be discussed in more depth later. Additionally, the average pooling layer concatenates several 2D and 3D convolutional layers with 8, 32, and 64 filters before forwarding them to the output layer and then to the output layer.

• The article’s primary contribution is optimizing hyperparameters—learning rate, batch size, regularization methods, and dropout rates—using Keras Tuner, which significantly improves the suggested CNN approach’s performance and its ability to converge to satisfactory solutions and generalize effectively to new data.

The rest of the article consists of the following sections. Materials and methods are presented in “Materials and Methods”. In this section, it is explained how the proposed CNN model is structured to incorporate both 3D-CNN and 2D-CNN architectures for feature extraction from the input data. Experimental analysis, results and discussion are presented in “Experiments and Results”. In this section, the results of the methane, methanol, and diesel reforming prediction are given. Finally, conclusions are drawn in “Conclusions”. Here, some findings and conclusions are revealed.

## Materials and Methods

Recent developments in ML and deep learning have led to the introduction of various neural network architectures (Wang et al., 2021). CNNs are a favorite among these algorithms because of their excellent effectiveness in challenging estimating tasks (Zhou et al., 2022). By using artificial feature extraction, this suggested CNN model, unlike existing ML techniques, automatically reveals hidden characteristics in the data (Abdalla et al., 2021; Mohamed, 2019; Moghara & Hamiche, 2020).

**Figure 1:**
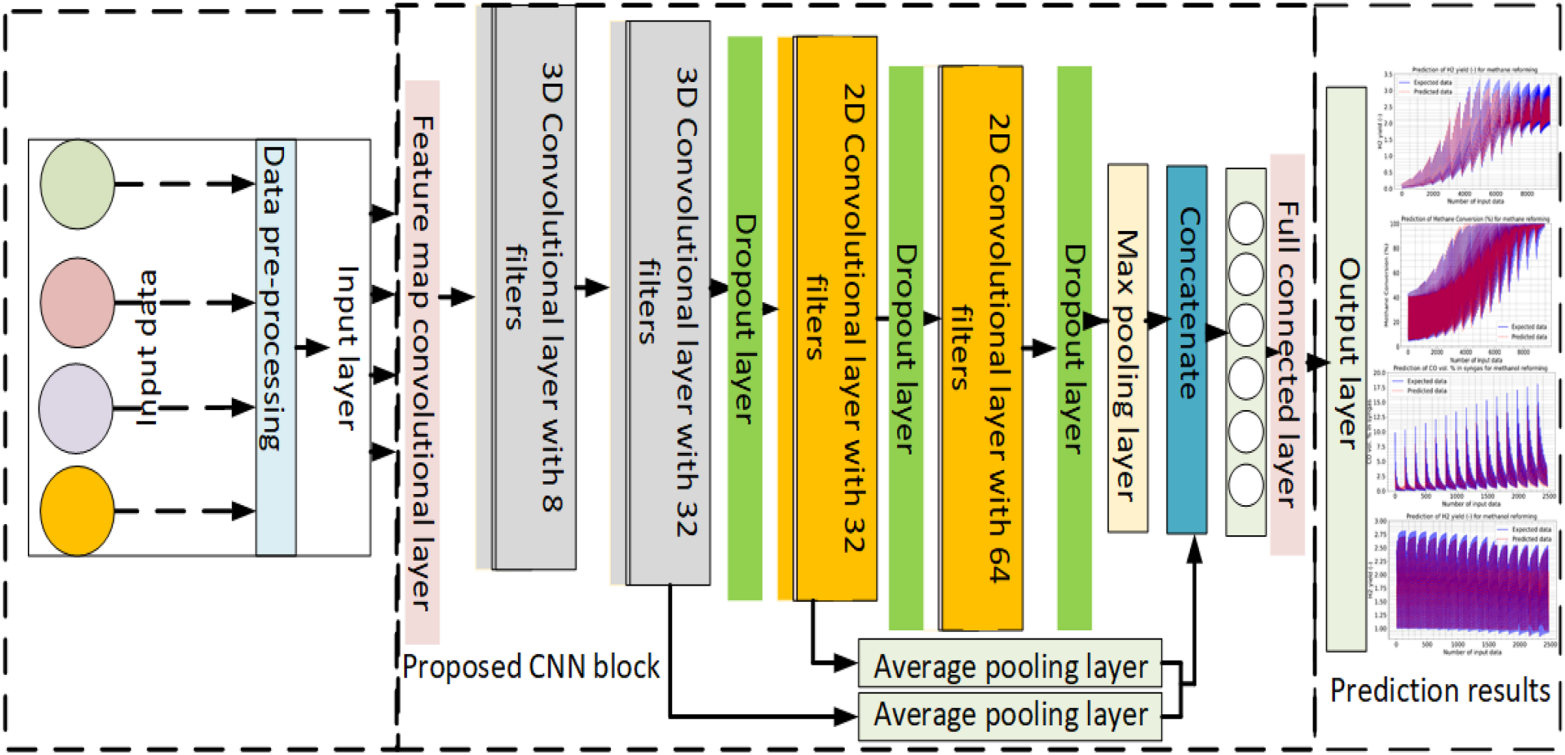
The developed CNN model for methane, methanol, and diesel reforming processes.

It has been employed the reformer temperature, reformer pressure, steam-to-methane ratio (-), oxygen to methane ratio (-) for estimating the methane reforming, reformer temperature, steam-to-methanol ratio, and oxygen-to-methanol ratio for the methanol-reforming, reformer temperature, steam to diesel ratio, oxygen to diesel ratio for diesel reforming. Following the completion of the preprocessing, these data are utilized as inputs for the suggested method. Figure 1 presents the proposed CNN model for methane, methanol, and diesel reforming prediction.

To execute data normalization, some outliers are eliminated from the initially collected data and replaced with the mean value of highly connected data values (Zhang et al., 2022). It is suggested that the reforming process be modeled after a strong and simpler CNN. The receptive field of this network, which is inspired by human membrane-bound neurons, responds to specific, constrained locations known as impulse responses. Until the visual area is completely developed, each neuron only partially covers this area. The convolution operations with CNN layers can be used to mathematically determine how each neuron responds to an impulse. By using some kernel matrices, the CNN learning approach can disclose crucial traits that can be applied to prediction. The link weights of the network are optimized using the backpropagation algorithm. The sliding window is used to create the layer’s fold (Yao, Xu & Ramezani, 2021). Floating-window weights and dot product weights are then added to produce a vector. On the original data, neurons with known rectified linear units (ReLUs) are subjected to the activation function according to Eq. (1).

(1) $$f\left( x \right) = {\rm max}\left( {x,0} \right)$$

The result is further scaled down using the max pooling layer (Yao, Xu & Ramezani, 2021). After network activation and CNN tuning, the prediction based on internal weights is optimized using the gradient descent approach, a method of minimization based on entropy loss. The cost function for this is shown by Eq. (2)

(2) $$L = \mathop \sum \nolimits_{j = 1}^N \mathop \sum \nolimits_{i = 1}^M - d_j^{\left( i \right)}logz_j^{\left( i \right)}$$

where ${z_j}$ stands for the planned and observed output vectors for the class number $M$, and ${d_j}$ = (0, …, 0, ${\rm \; }1,\mathop \to \limits^k \ldots 1$, 0, …, 0). Equation (3) produces a softmax function for the CNN

(3) $$z_j^{\left( i \right)} = \displaystyle{{{e^{{f_j}}}} \over {\mathop \sum \nolimits_{i = 1}^M {e^{{f_i}}}}}$$

where a weight penalty is used to modify the behavior of the final loss function $\; L$ specified in Eq. (4)

(4) $$L = \mathop \sum \nolimits_{j = 1}^N \mathop \sum \nolimits_{i = 1}^M - d_j^{\left( i \right)}logz_j^{\left( i \right)} + \displaystyle{1 \over 2}\mathop \sum \nolimits_K \mathop \sum \nolimits_L W_{k,l}^2$$

where ${W_k}$ and $K$ stand for the total number of layers and the weight connection in layer $l$, respectively.

In the designed CNN model, both 3D-CNN and 2D-CNN models are combined to extract features from the input data. The overall architecture of the model is illustrated in Fig. 1. In the designed CNN model, the same input data are received in both 2D and 3D formats to facilitate feature extraction. This means that the input data, which represent the characteristics of the fuel cell reforming process, are provided in a manner compatible with both 2D-CNN and 3D-CNN architectures. Specifically, the input data may be structured in a way that allows for the application of 2D convolutions across individual 2D slices or frames of the data, while also enabling the utilization of 3D convolutions to capture spatial and temporal dependencies across the entire volumetric data. By accommodating both 2D and 3D formats, the CNN model can effectively extract features from the input data, leveraging the strengths of each architecture to enhance the prediction accuracy of the reforming process. The preprocessing step is performed on the input data before it is introduced into the feature map convolutional layer (Carapellucci & Giordano, 2020). This preprocessing step could involve tasks such as normalization, resizing, or any other necessary data transformations to prepare the input for the CNN model. This step, performed prior to entering the feature map convolutional layer, typically involves tasks such as normalization, resizing, or any other necessary transformations to ensure that the input data is in a suitable format and scale for the CNN model. Normalization helps to standardize the input data, resizing adjusts the dimensions to match the network’s input size requirements, and other transformations may include data augmentation techniques to increase the diversity of the training dataset or to enhance model robustness. Overall, preprocessing ensures that the input data is properly formatted and optimized for effective training and inference within the CNN architecture. After preprocessing, the data are passed through a series of 3D convolutional layers. In this specific model, there are eight 3D convolutional layers. Each layer applies a set of filters to the input data to detect various features. The number of output channels or feature maps that each layer generates is determined by the number of filters in that layer. The 3D convolutional layers are designed to handle the input matrix with two input parameters and depth. The input matrix represents the 3D nature of the data, such as a volumetric image or a sequence of 3D frames. However, to combine the 3D-CNN and 2D-CNN models, the 3D input matrix is transformed into a 2D layer. In the prediction of reforming processes for various fuels like methane, methanol, and diesel within full cells, both 3D-CNN and 2D-CNN models find applications, albeit with differences in their approaches. For complex, volumetric reactions occurring within full cells, 3D-CNN models are particularly advantageous. They excel in capturing intricate spatial relationships within the fuel cell structure and dynamic changes in reforming behavior over time. This makes them well-suited for predicting reforming reactions where understanding the three-dimensional nature of the process is crucial. Conversely, 2D-CNN models are typically employed when dealing with spectroscopic or two-dimensional representations of the reforming process, such as data obtained from sensors or images. While not as adept at capturing the full volumetric dynamics, 2D-CNN models can still provide valuable insights into reforming behavior and make predictions based on features extracted from two-dimensional data. The choice between 3D-CNN and 2D-CNN models depends on the specific requirements of the prediction task, the availability of data, and computational considerations, all aimed at achieving accurate and efficient predictions of fuel cell reforming processes across different fuels. The transformation from a 3D input matrix to a 2D layer could be achieved through operations like reshaping or flattening the input. This process allows the subsequent layers to operate on a 2D representation of the data. By transforming the input, the model can leverage both the spatial and temporal information present in the 3D data while utilizing the advantages of the 2D convolutional layers. Following the 3D convolutional layers, the transformed 2D layer can be processed through additional layers in the model, which could include 2D convolutional layers, pooling layers, fully connected layers, or any other suitable architecture for the given task. In summary, the designed CNN model combines 3D-CNN and 2D-CNN models. The input data undergoe preprocessing and is then fed into a feature map convolutional layer. Subsequently, the data are processed through 8 3D convolutional layers, and the 3D input matrix is transformed into a 2D layer to facilitate the combination of the 3D and 2D aspects of the data.

To prevent overfitting, the 3D and 2D convolutional layers were bridged with the dropout layer. In the 2D convolution layer, comparatively 32 and 64 filters combine the characteristics between input parameters. Additionally, two dropout layers are employed before and after the 2D layers. Following additional processing with the second 3D convolution layer, a 3D matrix output is produced that merges the data for methane, methanol, and diesel. The max pooling layer receives 2D matrices from the 3D convolution layers. Following each 2D convolution layer, the recovered matrices of the feature information are sent to the concatenate layer *via* the jump link, as illustrated in Fig. 1. Additionally, two average pooling layers are utilized to integrate and in the CNN network, there are two 3D convolution layers and one 2D layer to reduce computation and address the issue of feature redundancy. Additionally, the fully connected layer is not reached until the combination of feature matrices of the same size. The output layer is where the methane, methanol, and diesel data estimation are obtained after these fully connected layers.

In this work, it has been evaluated and validated the suggested technique using the RMSE, MAPE, MAE, and R^2^ metrics. The difference between samples of data or both the actual values and those predicted by an estimator or a model for all data values is often calculated using the RMSE. The RMSE stands for root mean square error, often known as the quadratic mean of the differences between expected values and actual values. Equation (5) is used to calculate the RMSE. The more accurate the data prediction is found, the lower the RMSE value. Therefore, the RMSE values must be reduced if the proposed model’s high performance is to be desired. The proposed CNN approach is employed to find and choose the number of layers and related hidden nodes. The closer to zero the RMSE, MAPE, and MAE values are, the better the prediction performance

(5) $$RMSE = \sqrt {\displaystyle{{\mathop \sum \nolimits_{t = 1}^T {{\left( {x_t^p - x_t^e} \right)}^2}} \over T}}$$

where $T$ is the number of samples, $x_t^p$ and $x_t^e$ are the predicted and expected value at sample $t$, respectively. The issue of estimation errors dampening each other in both the positive and negative directions is avoided by MAE. The MAE calculation is provided in Eq. (6).

(6) $$MAE = \displaystyle{1 \over T}\mathop \sum \nolimits_{t = 1}^T \left| {x_t^p - x_t^e} \right|$$

The difference between the actual and anticipated numbers is calculated as a percentage error using MAPE. It also depends on the relationship between error and true value, which makes the forecast result’s accuracy easier to see. The formula for calculating the MAPE is shown in Eq. (7).

(7) $$MAPE = \displaystyle{1 \over T}\mathop \sum \nolimits_{t = 1}^T \left| {\displaystyle{{x_t^p - x_t^e} \over {x_t^e}}} \right|$$

To determine how closely the parameter data follow the estimated regression line, it has been additionally calculated R^2^ called as coefficient of determination. An indicator of how well a model fits the data is R^2^. It is computed by dividing the entire sum of squares by the sum of the squares of the discrepancies between the observed and anticipated values. A result is a number in the range of 0 and 1, where 0 means that no variations in the data are explained by the model and 1 means that all variations in the data are explained by the model. The coefficient of determination is another name for R^2^. The performance improves as R^2^ increases. Keep in mind that R^2^ performs best at a value of 1. We calculate the R^2^ as given in Eq. (8).

(8) $${R^2} = 1 - \displaystyle{{\mathop \sum \nolimits_{t = 1}^T {{\left( {x_t^e - x_t^p} \right)}^2}} \over {\mathop \sum \nolimits_{t = 1}^T {{\left( {x_t^e - \displaystyle{{\mathop \sum \nolimits_{t = 1}^T x_t^e} \over T}} \right)}^2}}}$$

The suggested 2D and 3D CNN algorithms are executed in only one for loop is used for an input data sample, significantly increasing the algorithm’s complexity. Although the LSTM and CNN structures, which employ more nested neurons, layers, and complexity tailored for image processing and recognition, use more nested neurons, it is believed that the proposed technique produces results that can make more efficient and accurate predictions.

## Experiments and results

### Dataset in this research

The input parameters in the dataset were selected while considering the most critical operational parameters of the reformers, which are reformer temperature, steam-to-fuel ratio, and oxygen-to-fuel ratio. In addition, the reformer pressure was used as an input parameter for methane reforming because methane conversion strongly depends on the reformer pressure. On the other hand, the output parameters in the dataset were selected while considering widely used fuel cells in the market, which are PEMFCs, HT-PEMFCs, and SOFCs. Hydrogen and carbon monoxide contents in the syngas are two most important parameters which affect the performance of SOFCs (Harun, Tucker & Adams, 2016, 2017), PEMFCs (Sahebdelfar & Ravanchi, 2022). Roughly estimation of output power of a fuel cell is also possible while using hydrogen and carbon monoxide content in the syngas.

The dataset was obtained by using Aspen Plus simulation software. RGibbs reactor in Aspen Plus with Peng-Robinson equation of state was used to estimate the syngas composition of methane, methanol, and diesel reforming. RGibbs reactor estimates the reaction components by using Gibbs free energy minimization method. The other software can be also used for syngas composition estimation by employing Gibbs free energy minimization method (Caglar, Tavsanci & Biyik, 2021). Indeed, the Gibbs free energy minimization method does not consider reaction kinetics and type of the catalysts. However, this method is widely used in the literature to estimate the performance of reformatted gas fueled fuel cell systems. Therefore, the Gibbs free energy minimization method was selected for this study to create the dataset.

Reforming temperature, steam-to-fuel ratio, and oxygen-to-fuel ratio were used as input parameters to develop the ML method. The pressure was also considered as an input parameter for methane reforming. Hydrogen yield and carbon monoxide vol.% in the syngas were estimated by using the proposed ML technique. In addition, methane conversion was estimated for methane reforming because methane reforming is thermodynamically limited, and its conversion strongly depends on the operation conditions of the reformer.

As a result of the processes and analyzes, we created the dataset. The dataset consists of an unprecedented and previously unused Excel data file prepared separately for methane, methanol, and diesel reforming. For methane reforming, the input parameters are reformer temperature in °C, pressure in atm, steam to methane ratio (-), and O_2_ to methane ratio (-), and output parameters are H_2_ yield (-), CO vol. % in syngas, and methane conversion (%). The data in all parameters for methane reforming is 9,450 in total. For methanol reforming, the input parameters are reformer temperature in °C, steam to methanol ratio (-), and O_2_ to methanol ratio (-), and output parameters are H_2_ yield (-) and CO vol. % in syngas. The data in all parameters for methanol reforming are 2,475 in total. For diesel reforming, the input parameters are reformer temperature in °C, steam to diesel ratio (-), and O_2_ to diesel ratio (-), and output parameters are H_2_ yield (-) and CO vol. % in syngas. The data in all parameters for diesel reforming is 22,275 in total. For methane reforming, the reformer temperature and pressure change from 300 to 1,000 °C, and 1 to 50 atm, respectively, while the steam-to-fuel ratio and oxygen-to-fuel ratio vary from 1 to 4 and 0.084 to ~0.8, respectively. On the other hand, the temperature, steam-to-fuel, and oxygen-to-fuel ratios range from 250 to 600 °C, 0.5 to 3, and 0.084 to ~1, respectively for methanol reforming while these values are equal to 350 to 1,000 °C, 16 to 32, and 7.35 to ~16.2 for diesel reforming.

Data analysis has been performed in the experiments, modeling, and training of the data. In the study, the Keras Tuner method is used in Python to determine the best values of these hyperparameters. With the Keras Tuner, batch size, learning rate, time steps, optimization method selection, convolution layer number and type, and other similar hyperparameters are determined thanks to this improvement method. As a result of this optimization, although the learning rate, testing rate, and validation rate were 79.86%, 10.05%, and 10.09%, respectively, for each data set, it has been used 80% of the total data for training, 10% for testing, and the final 10% for validation. It has been randomly selected these numbers from the dataset so that the accuracy of the results is not distorted. The effectiveness of scaling and normalization techniques is not compromised by randomly selecting numbers from the dataset. As long as the selection process is unbiased and representative of the dataset as a whole, the resulting accuracy of the analysis or training should not be distorted. When preparing data, an interpolation operation is used to fill in missing values in the data frame or series. Also, the data are scaled from 0 to 1 so that all models can be properly trained before training. After the training, the data have been converted back to their original values to compare and visually present the estimated and actual values of the data. Some statistical data of methane, methanol, and diesel reforming in the dataset are presented in Table 1.

**Table 1:** The parameter data values of the dataset used in the study.

Type of fuel reforming	Output parameter	Maximum	Minimum	Average
	H_2_ yield (−)	3.35834692	0.01702854	1.290020304
Methane reforming	CO vol. % in syngas	22.4848289	0.000327662	3.668477641
	Methane conversion (%)	99.9999069	4.64798915	60.23560287
Methanol reforming	H_2_ yield (−)	2.81794461	0.8424552	1.791084915
	CO vol. % in syngas	18.0649519	0.028099713	2.193244143
Diesel reforming	H_2_ yield (−)	32.9551993	8.85806686	19.81499573
	CO vol. % in syngas	2.40116276	0.277111735	0.917752861

### Experimental setup

To carry out the study’s applications, a Windows 10 computer with an Intel R-Core i5-4210 processor, 6 GB of RAM, and an Nvidia Geforce 4 GB graphics card, is used. We used the Python programming and its libraries. Two of the four convolutional layers of the deep CNN that utilized are 3D, while the other two are 2D. The CNN model consists of two average pooling layers, a max pooling layer, and three dropout layers. The suggested model has a fully connected layer that comes before the output layer. After normalization, each layer applies a ReLU function as the activation function to the input data. The batch size and the maximum number of epochs for the proposed CNN model’s training are set to 64 and 100 as a result of the tuner, respectively. The Adam optimizer is also employed in the learning process for adaptive optimization. The experiments in this study have been run algorithms with a Keras Tuner and the best learning rate has been found at 0.001. In other words, the learning rate has been determined as adaptive in the study. It has been observed that a learning rate smaller than this determined value worsens the algorithm performance, and a larger value tends to overlearn the model.

### Performance results and discussion

In this section, the performance outcomes of the proposed CNN model are discussed for predicting methane, methanol, and diesel reforming. Numerous AI algorithms are suggested in the experiments for foreseeing the reforming of methane, methanol, and diesel. In terms of the performance metrics RMSE, MAPE, MAE, and R^2^, the proposed CNN approach has been compared with other algorithms such as SVM (Chen & Lee, 2015), ANN (Hamzaçebi, 2008), LSTM (Bouktif et al., 2018), LSTM-GRU (Saini et al., 2020), Bi-LSTM (Kim & Moon, 2019), and standard CNN (Mehtab & Sen, 2020).

a) Support vector machines (SVM) (Chen & Lee, 2015): A supervised learning algorithm utilized for regression and classification issues is SVM. In order to divide classes or estimate continuous values, it builds a hyperplane or collection of hyperplanes in a high-dimensional space. The margin between the training data points and the decision border is what SVM seeks to maximize. By using several kernel functions, including linear, polynomial, radial basis function (RBF), and sigmoid, it can handle both linear and nonlinear data.

b) Artificial neural network (ANN) (Hamzaçebi, 2008): A class of machine learning models known as ANNs is motivated by the design and operation of biological neural networks. It is made up of layers of artificial neurons or nodes that are connected to one another. Recurrent, feedforward, or a mix of the two can be found in ANN models. Through a technique known as backpropagation, they learn from data. In this process, the model modifies the weights and biases among nodes to reduce the discrepancy between expected and actual outputs. Many tasks, such as pattern recognition, regression, and classification, can be handled by ANN.

c) Long short-term memory (LSTM) (Bouktif et al., 2018): Recurrent neural networks (RNNs) of the long-term dependency (LSTM) type were created to solve the vanishing gradient issue and identify long-term relationships in sequential data. Memory cells and specific gates are introduced, enabling the long-term storing, updating, and retrieval of data. When it comes to modeling sequential patterns, LSTM works especially well in applications like time series analysis, speech recognition, and natural language processing.

d) Long short-term memory-gated recurrent unit (LSTM-GRU) (Saini et al., 2020): LSTM-GRU is a hybrid model that combines the strengths of LSTM and GRU (Gated Recurrent Unit). GRU is another type of RNN that simplifies the architecture of LSTM by combining the input and forget gates into a single “update” gate. LSTM-GRU aims to balance the model’s ability to capture long-term dependencies (LSTM) with computational efficiency (GRU). It has been found to perform well in various sequence modeling tasks.

e) Bidirectional LSTM (Bi-LSTM) (Kim & Moon, 2019): Bi-LSTM is an extension of the LSTM model that processes input sequences in both forward and backward directions. By utilizing information from both past and future contexts, Bi-LSTM captures more comprehensive representations of the input sequence. It is commonly used in tasks where the current prediction depends on both the preceding and succeeding elements, such as machine translation, sentiment analysis, and named entity recognition.

f) ConvNet, another name for standard CNN (Mehtab & Sen, 2020), is a deep learning model that is specifically made to interpret structured input that resembles a grid, including time series data and photographs. CNNs are made up of completely linked, pooling, and many convolutional layers. In order to extract local patterns and features from the input data, the convolutional layers apply filters to it. By reducing spatial dimensions, pooling layers extract the most notable features. In computer vision applications including object identification, image segmentation, and image classification, CNNs have demonstrated remarkable efficacy (Salam & Hibaoui, 2021; Peksen & Spliethoff, 2023; Li & Zhou, 2023; Oladosu et al., 2024).

#### The prediction of the methane reforming

It has been used reformer temperature, reformer pressure, steam-to-methane ratio (-), and oxygen-to-methane ratio (-) as the input parameters for methane reforming predictions. By processing these input parameter data, the predicted outputs of the hydrogen yield (produced hydrogen mole/consumed methane mole), carbon monoxide vol. % in syngas, and methane conversion (%), are found. After that, it has been compared the actual H_2_, CO, and methane conversion data with their estimated values.

**Figure 2:**
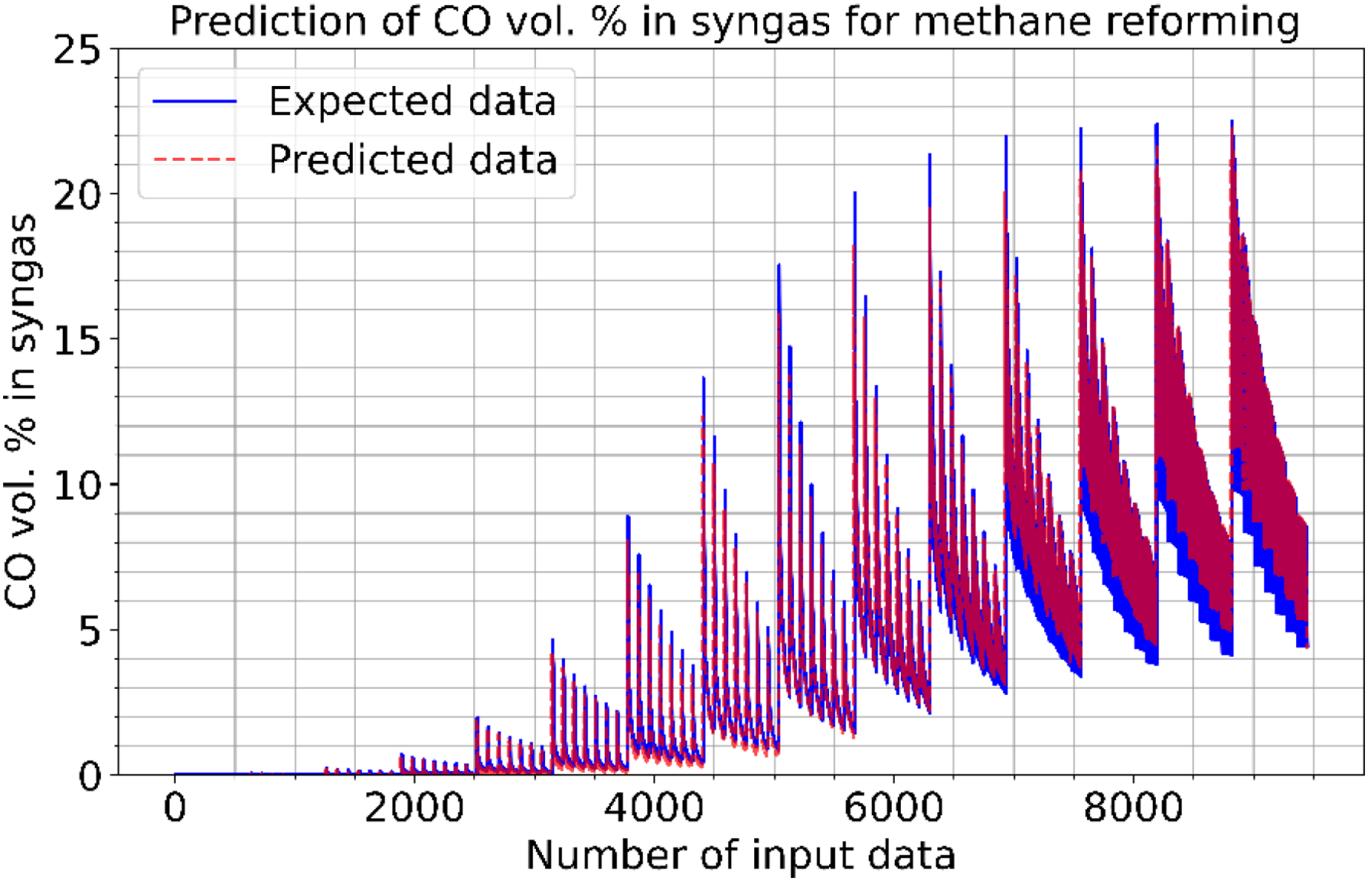
Prediction of CO vol. % syngas for methane reforming.

**Figure 3:**
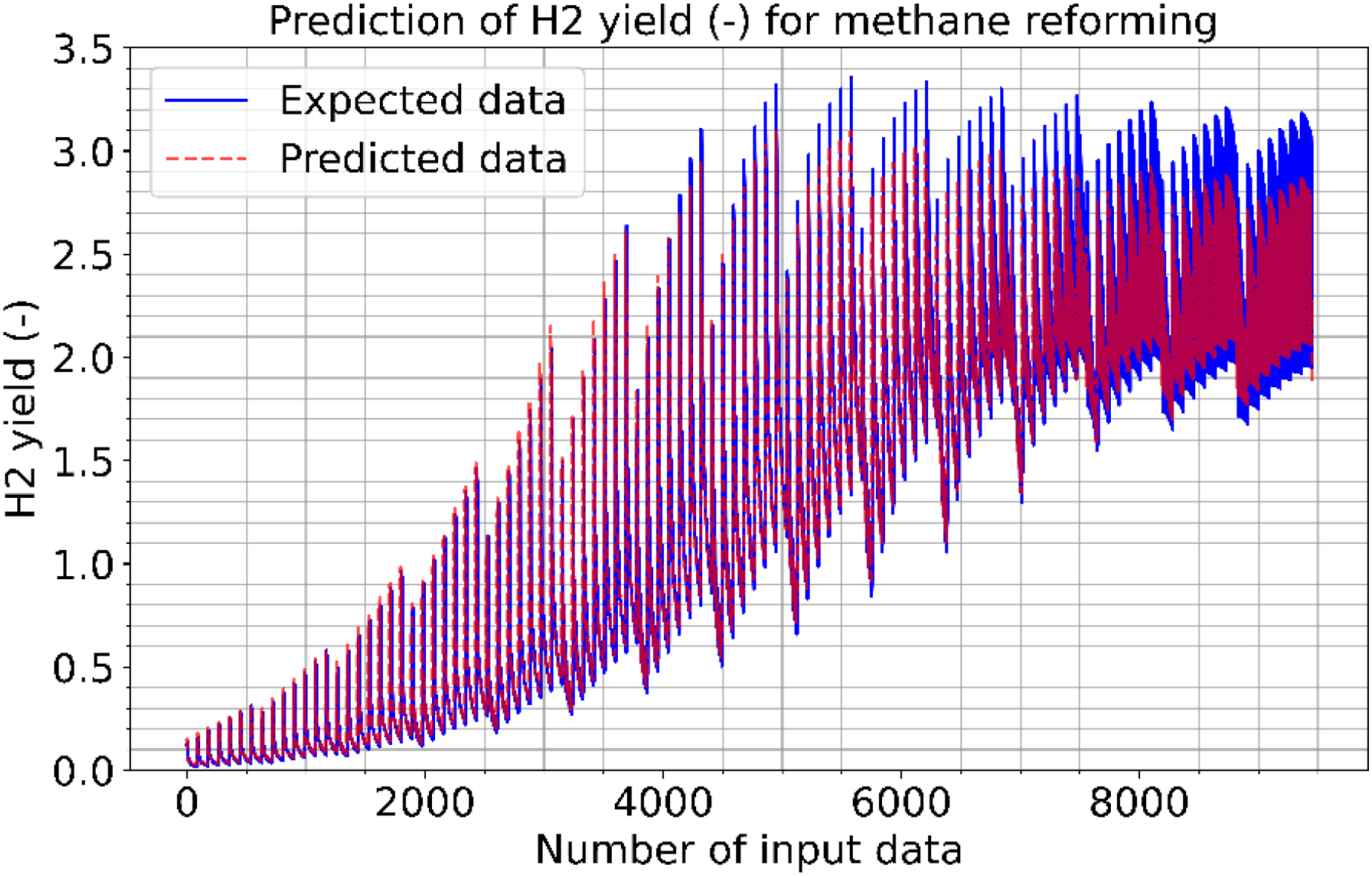
Prediction of H2 yield (−) in molconsumed methane (mol) for methane reforming.

**Figure 4:**
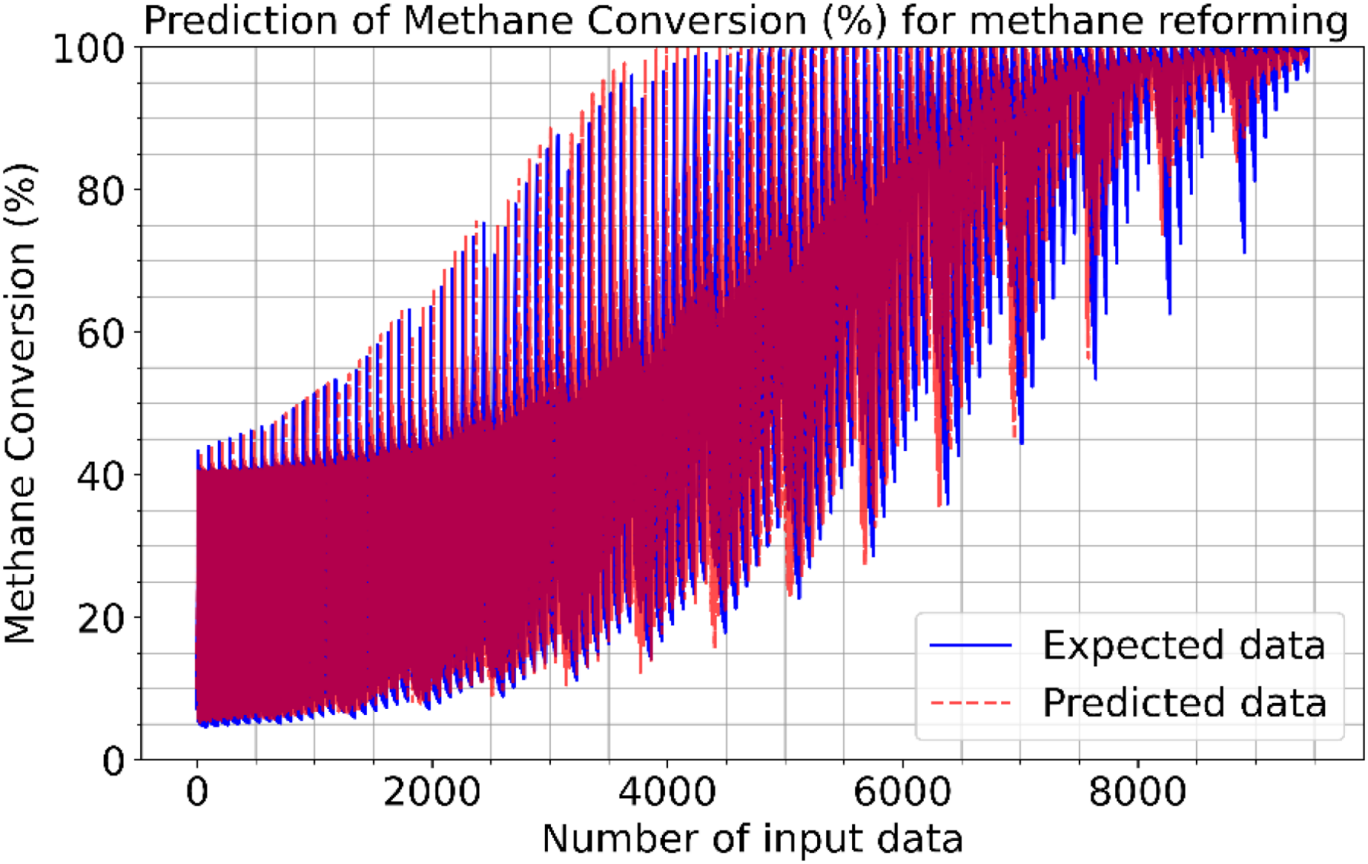
Prediction of methane conversion (%) for methane reforming.

**Figure 5:**
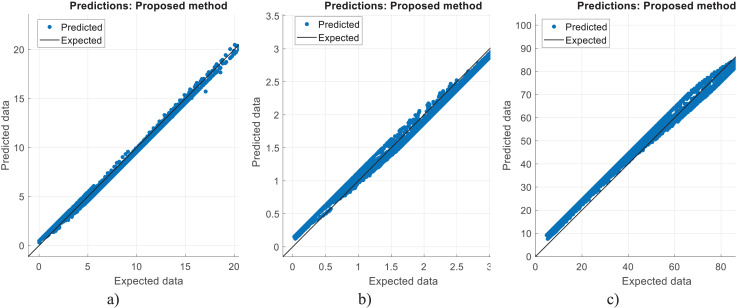
Prediction errors for methane reforming. (A) CO vol. % syngas. (B) H2 yield (−) in molconsumed methane (mol). (C) Methane conversion (%).

Figures 2–4 illustrate the prediction of CO vol. % syngas, H_2_ yield (-) in mole, and methane conversion (%) for methane reforming, respectively. The values for the estimated CO vol. in syngas are quite similar to those for the actual (expected). Figures 2–4 illustrate how the CO syngas, H_2_ yield, and methane conversion values and the absolute difference between the estimated value and the actual value change as the amount of data rises. This demonstrates that the error rates are progressively dropping. Carbon monoxide values that can adjust to the learning vector values and that can be provided as input values in parallel with normalization have been developed for use in the proposed CNN architecture. Figure 5 shows the prediction errors for methane reforming data in scattered points.

In terms of error metrics, Table 2 compares the proposed method with other methods based on methane reforming. For all methods, the best success was achieved in CO vol. % syngas, while the worst success was experienced in methane conversion. The suggested CNN method yielded the best results, whereas the SVM approach performed the worst in the test results. The proposed CNN is compared with all other methods. As can be seen from the performance results, the SVM method showed the worst performance for the dataset created and used. Because the error rates (such as RMSE, MAPE) are the highest and the R^2^ rate is the highest.

**Table 2:** The comparison of the proposed method with other methods based on methane reforming.

Methods	Error metrics	Output parameters		
		Carbon monoxide vol. % in syngas	H_2_ yield (−) in mole	Methane conversion (%)
SVM (Chen & Lee, 2015)	RMSE	11.72	12.64	13.42
	MAE	10.46	11.49	11.94
	MAPE	9.38	9.67	10.14
	R^2^	0.9678	0.9629	0.9576
	ACT (s)	2.452	2.611	2.795
ANN (Hamzaçebi, 2008)	RMSE	11.35	12.16	12.67
	MAE	10.17	11.09	11.62
	MAPE	9.08	9.24	9.84
	R^2^	0.9762	0.9701	0.9649
	ACT (s)	2.357	2.486	2.674
LSTM (Bouktif et al., 2018)	RMSE	10.54	11.67	12.35
	MAE	9.28	10.37	11.12
	MAPE	7.69	8.42	9.27
	R^2^	0.9823	0.9778	0.9713
	ACT (s)	1.993	2.278	2.358
LSTM-GRU (Saini et al., 2020)	RMSE	10.28	11.14	11.68
	MAE	8.45	9.89	10.56
	MAPE	6.76	7.39	8.17
	R^2^	0.9878	0.9847	0.9804
	ACT (s)	1.856	1.984	2.247
Bi-LSTM (Kim & Moon, 2019)	RMSE	8.89	9.52	10.16
	MAE	6.49	7.83	8.34
	MAPE	4.39	4.91	5.69
	R^2^	0.9917	0.9896	0.9859
	ACT (s)	1.652	1.761	1.945
Standard CNN (Mehtab & Sen, 2020)	RMSE	4.58	5.48	6.45
	MAE	2.64	3.52	4.28
	MAPE	1.86	2.41	3.39
	R^2^	0.9961	0.9946	0.9903
	ACT (s)	1.598	1.627	1.756
Proposed method	RMSE	3.17	4.49	5.31
	MAE	1.68	1.99	2.68
	MAPE	0.86	1.28	1.85
	R^2^	0.9989	0.9964	0.9915
	ACT (s)	1.496	1.564	1.627

#### The prediction of the methanol reforming

It has been used reformer temperature, steam-to-methanol ratio, and oxygen-to-methanol ratio as the input parameters for methanol reforming predictions. By processing these input parameter data, the estimated outputs of the hydrogen yield (produced hydrogen mole/consumed methanol mole) and carbon monoxide vol. % in syngas, are found. Next, the actual H_2_ and CO data are compared with their estimated values.

**Figure 6:**
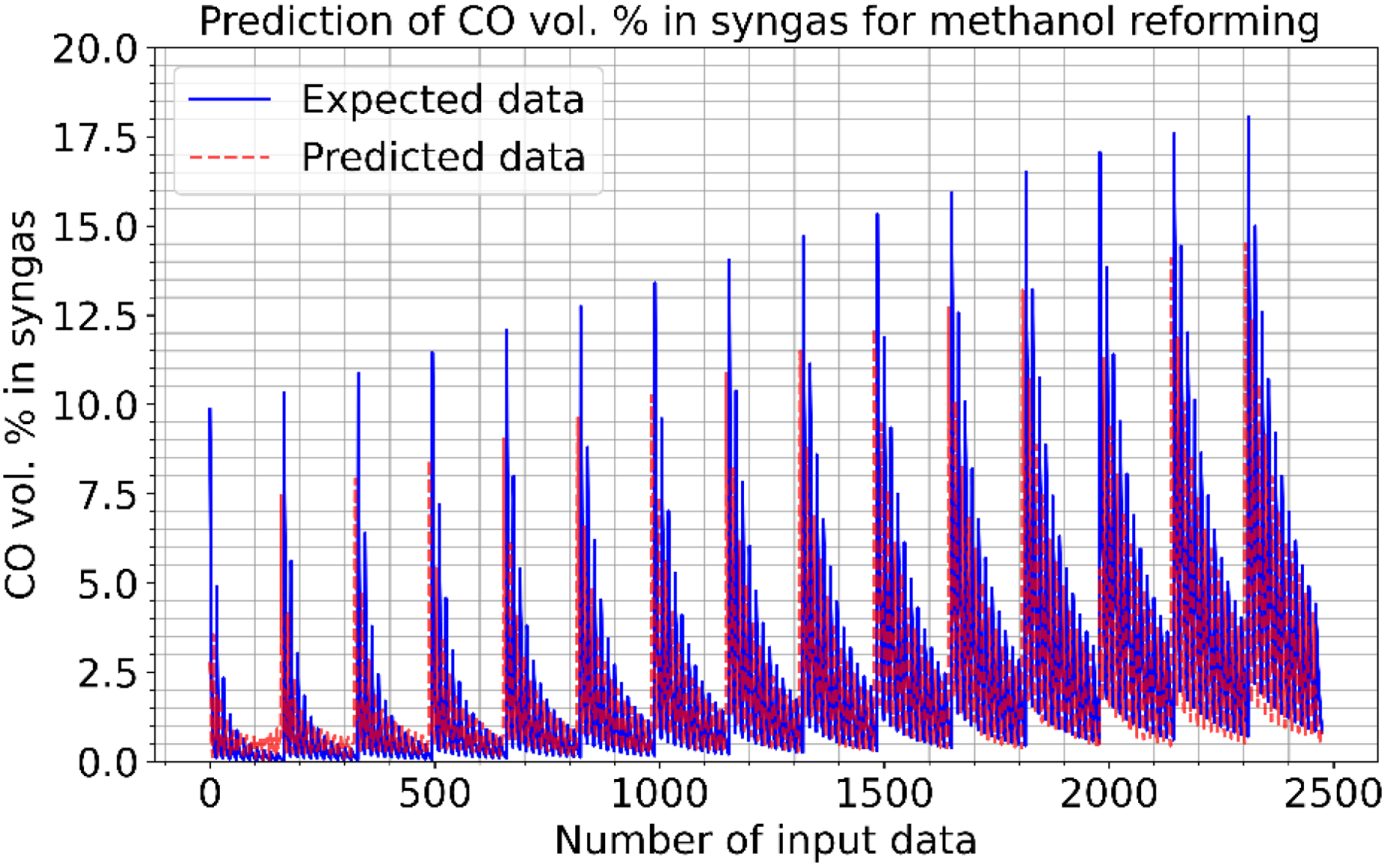
Prediction of CO vol. % syngas for methanol reforming.

**Figure 7:**
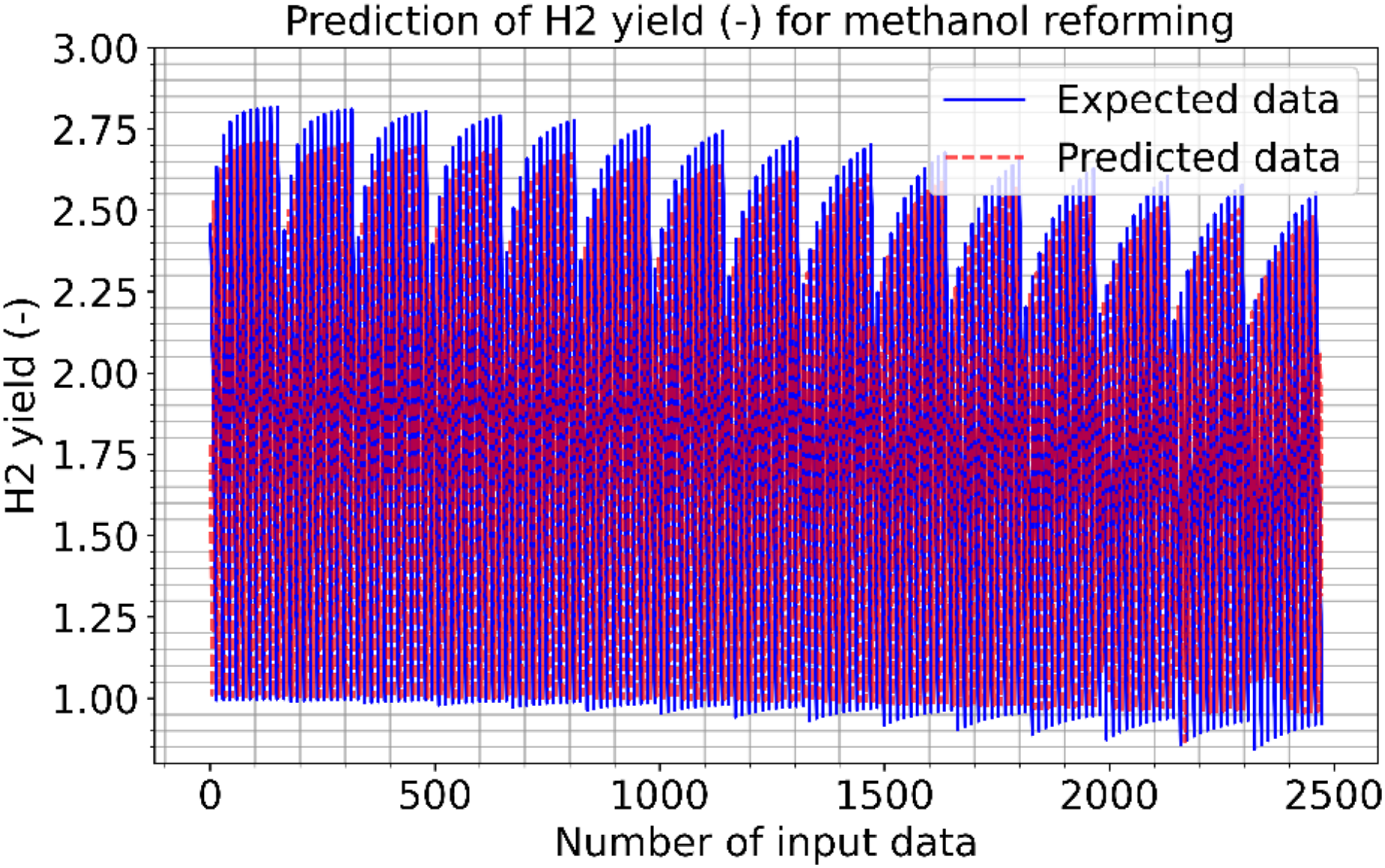
Prediction of H2 yield (−) in molconsumed methane (mol) for methanol reforming.

**Figure 8:**
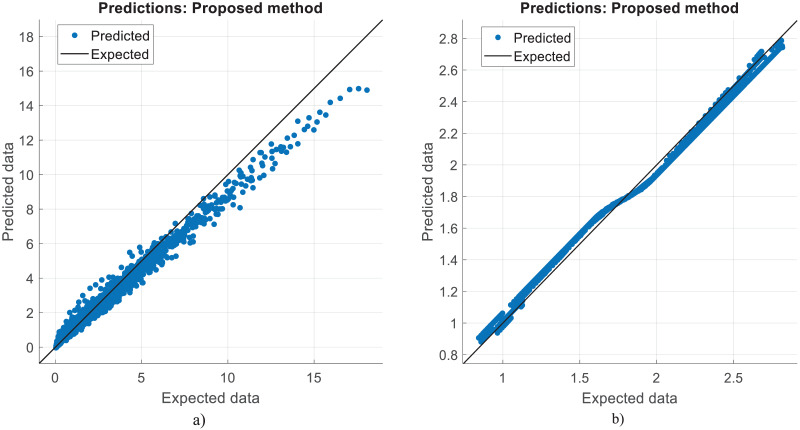
Prediction errors for methanol reforming. (A) CO vol. % syngas. (B) H2 yield (−) in molconsumed.

Figures 6 and 7 show the estimated results of CO vol. % syngas and H_2_ yield (-) in mole for methanol-reforming, respectively. From the results, Once the proposed CNN is carried out, it gives that there is a minimal difference between the expected values and the expected values. Hence, the data prediction performance is also quite good. As seen in Fig. 6, as the number of data increase, the CO value increases and the absolute value between the estimated value and the actual value decreases. This shows that the error rates are gradually decreasing. As can be seen in Fig. 7, the value of the H_2_ product decreases as the number of data increase. The absolute value between the data predicted value and the actual value also decreases. In other words, it is understood that the error rates are gradually decreasing. Figure 8 shows the prediction errors for methanol reforming data in scattered points.

Table 3 shows the comparison of the presented method with other methods based on methanol-reforming. In the experiments, CO vol.% syngas had a better success rate than H_2_ yield. In the test results, the proposed CNN algorithm outperformed the SVM technique, which had the poorest performance. Additionally, LSTM, LSTM-GRU, Bi-LSTM, standard CNN, and proposed CNN all outperform the ANN technique in all predictions. In CO prediction, the proposed CNN outperforms the SVM with an R^2^ of 0.9934 as opposed to 0.9347. Even though the LSTM method performed better than other algorithms, it still performed worse than the proposed CNN. This indicates that the proposed CNN’s RMSE, MAE, and MAPE error rates are lower than those of the other learning techniques. It is obvious that better R^2^ scores are obtained with the proposed CNN than the alternative approaches. The proposed CNN has a ratio of 6.12, 4.23, 2.89, and 0.9872 when compared to standard CNN, which has RMSE, MAE, MAPE, and R^2^ for H_2_ yields of 9.33, 6.96, 4.55, and 0.9814, respectively. The proposed approach produced 5.62, 3.18, 2.26, and 0.9934 for RMSE, MAE, MAPE, and R^2^, respectively, in the conversion of methane. It is obvious that the success in estimating CO is higher than that in estimating H_2_. The ACT of the proposed CNN is 0.648 and 0.756, for CO vol. % in syngas and H_2_ yield (-) in mole, respectively.

**Table 3:** The comparison of the proposed method with other methods based on methanol.

Methods	Error metrics	Output parameters	
		Carbon monoxide vol. % in syngas	H_2_ yield (−) in mole
SVM (Chen & Lee, 2015)	RMSE	18.64	19.35
	MAE	16.85	17.39
	MAPE	14.63	15.46
	R^2^	0.9347	0.9312
	ACT (s)	1.952	2.235
ANN (Hamzaçebi, 2008)	RMSE	17.35	18.52
	MAE	15.25	16.63
	MAPE	12.49	12.99
	R^2^	0.9451	0.9376
	ACT (s)	1.759	1.992
LSTM (Bouktif et al., 2018)	RMSE	16.29	17.66
	MAE	14.56	15.94
	MAPE	10.18	11.73
	R^2^	0.9539	0.9449
	ACT (s)	1.523	1.678
LSTM-GRU (Saini et al., 2020)	RMSE	15.64	16.95
	MAE	13.74	15.13
	MAPE	9.38	10.17
	R^2^	0.9668	0.9603
	ACT (s)	1.341	1.469
Bi-LSTM (Kim & Moon, 2019)	RMSE	13.12	14.28
	MAE	9.39	10.72
	MAPE	5.43	6.66
	R^2^	0.9714	0.9653
	ACT (s)	0.966	1.264
Standard CNN (Mehtab & Sen, 2020)	RMSE	7.26	9.33
	MAE	5.26	6.96
	MAPE	3.42	4.55
	R^2^	0.9892	0.9814
	ACT (s)	0.796	0.889
Proposed method	RMSE	5.62	6.12
	MAE	3.18	4.23
	MAPE	2.26	2.89
	R^2^	0.9934	0.9872
	ACT (s)	0.648	0.756

#### The prediction of the diesel reforming

It has been utilized reformer temperature, steam-to-diesel ratio, and oxygen-to-diesel ratio as the input parameters for diesel reforming predictions. By processing these input parameter data, the estimated outputs of the hydrogen yield (produced hydrogen mole/consumed diesel mole) and carbon monoxide vol. % in syngas, are found. Next, the actual H_2_ and CO data are compared with their estimated values.

**Figure 9:**
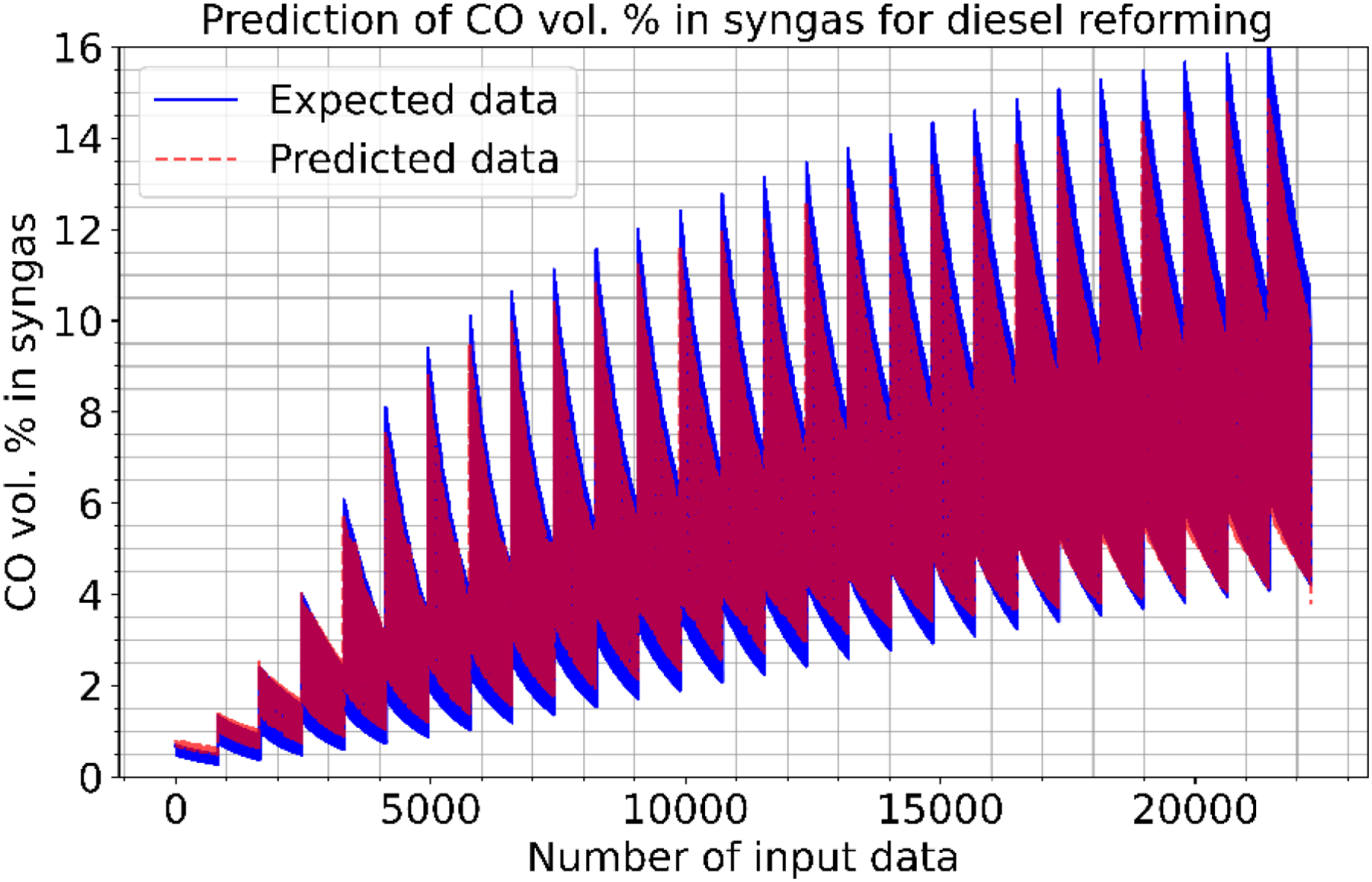
Prediction of CO vol. % syngas for diesel reforming.

**Figure 10:**
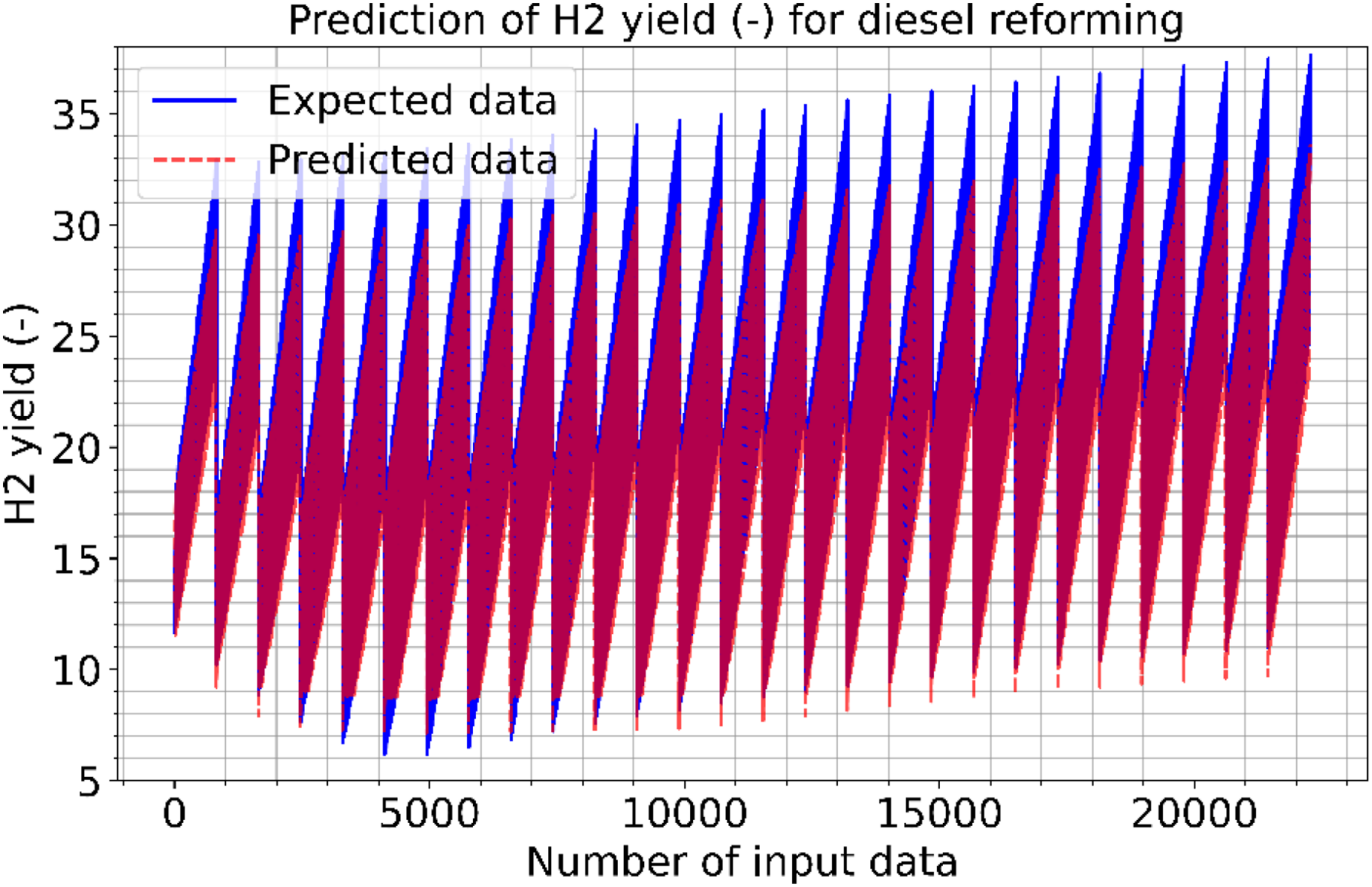
Prediction of H2 yield (−) in molconsumed methane (mol) for diesel reforming.

**Figure 11:**
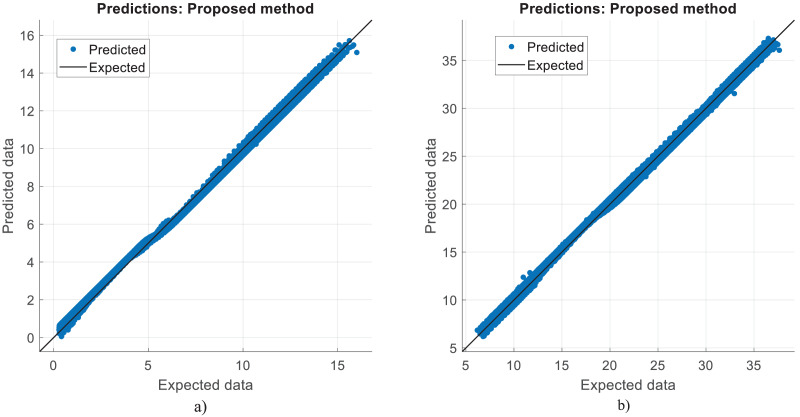
Prediction errors for diesel reforming. (A) CO vol. % syngas. (B) H2 yield (−) in molconsumed.

Figures 9 and 10 show the estimated results of CO vol. % syngas and H_2_ yield (-) in mole for diesel reforming, respectively. According to the findings, the diesel reforming estimation values are extremely near to the actual data. As a result, the diesel reforming data estimation performance is comparable to the estimations in terms of quality. Figure 11 shows the prediction errors for diesel reforming data in scattered points.

Table 4 shows the performance results of the proposed method and other methods based on diesel reforming. In the experiments, CO vol.% syngas had a better success rate than H_2_ yield in diesel reforming predictions. From the results, it has been understood that the proposed CNN algorithm outperformed the SVM technique that had the worst performance. LSTM, LSTM-GRU, Bi-LSTM, standard CNN, and proposed CNN all outperform the ANN method in the predictions. The proposed method performs better in CO prediction than the SVM, with an R^2^ of 0.9989 against 0.9827. While the LSTM method performed better than other methods, it still performed worse than the proposed method. This means that the RMSE, MAE, and MAPE error rates of the proposed CNN are lower than those of the other learning techniques. The proposed CNN has a ratio of 1.13, 0.74, 0.21, and 0.9962 when compared to standard CNN, which has RMSE, MAE, MAPE, and R^2^ of 1.69, 1.53, 1.26, and 0.9952 for H_2_ yields, respectively. The proposed CNN has a rate of 0.57, 0.23, 0.09, and 0.9989 when compared to standard CNN, which has RMSE, MAE, MAPE, and R^2^ of 9.33, 6.96, 4.55, and 0.9814 for CO prediction, respectively. The proposed approach produced 1.35, 1.13, 0.86, and 0.9976 for RMSE, MAE, MAPE, and R^2^, respectively, in the conversion of methane. The success in estimating CO is higher than that in estimating H_2_ yield. The ACT of the proposed CNN was 3.667 and 4.378, for CO vol. % in syngas and H_2_ yield (-) in mole, respectively. That is, from all the results, it has inferred that high performances are obtained at the prediction of reforming parameters in the experiments. Note that diesel reforming estimation error rates were obtained quite low compared to other methane and methanol estimations. Parallel to this, the R^2^ ratios were also high. This is because the number of diesel reforming data is higher than the others. By processing more data, the quality and success of learning have increased. However, the ACT of the algorithms is higher in diesel reforming than the others. This is due to the functioning of the algorithm and the long learning times of neurons in the CNN layers.

**Table 4:** The comparison of the proposed method with other methods based on diesel reforming.

Methods	Error metrics	Output parameters	
		Carbon monoxide vol. % in syngas	H_2_ yield (−) in mole
SVM (Chen & Lee, 2015)	RMSE	8.16	8.39
	MAE	7.25	7.64
	MAPE	5.39	5.89
	R^2^	0.9827	0.9756
	ACT (s)	5.233	5.746
ANN (Hamzaçebi, 2008)	RMSE	6.59	7.28
	MAE	5.13	6.42
	MAPE	3.14	3.75
	R^2^	0.9852	0.9815
	ACT (s)	4.951	5.547
LSTM (Bouktif et al., 2018)	RMSE	5.16	5.69
	MAE	3.43	4.03
	MAPE	2.24	2.86
	R^2^	0.9876	0.9823
	ACT (s)	4.740	5.354
LSTM-GRU (Saini et al., 2020)	RMSE	4.23	4.86
	MAE	3.11	3.67
	MAPE	1.38	1.88
	R^2^	0.9926	0.9853
	ACT (s)	4.583	4.954
Bi-LSTM (Kim & Moon, 2019)	RMSE	3.17	3.82
	MAE	2.22	2.91
	MAPE	1.02	1.41
	R^2^	0.9952	0.9912
	ACT (s)	4.395	4.678
Standard CNN (Mehtab & Sen, 2020)	RMSE	1.35	1.69
	MAE	1.13	1.53
	MAPE	0.86	1.26
	R^2^	0.9976	0.9952
	ACT (s)	3.961	4.496
Proposed method	RMSE	0.57	1.13
	MAE	0.23	0.74
	MAPE	0.09	0.21
	R^2^	0.9989	0.9962
	ACT (s)	3.667	4.378

Regarding the average computational time reported in Tables 2–4, the average computational time obtained from running 50 times is based on the study. To gain a better understanding of the reliability and consistency of the proposed method, the proposed method was executed 50 of times for each experiment and the variability of the results across those runs. This include metrics such as standard deviation and confidence intervals to provide insights into the stability and robustness of the proposed CNN method.

To analyze the characteristics of the proposed CNN method that contributed to its better results compared to other methods, a detailed examination of the experimental setup and results were performed. The specific characteristics that contributed to the superior performance of the proposed CNN method are included:

a) The design choices of the CNN architecture, such as the number and types of layers, the size of filters, the use of pooling, normalization layers, and any specific architectural innovations, played a role in the superior performance. The proposed CNN model have been better able to capture and represent the relevant features and patterns in the data compared to the other algorithms.

b) The preprocessing techniques applied to the data before feeding it into the CNN model have influenced the results. Appropriate data preprocessing steps, such as normalization, and feature scaling enhance the proposed model’s ability to learn and generalize from the data.

c) The process of tuning hyperparameters, such as learning rate, batch size, regularization techniques, and dropout rates using Keras Tuner contributed to the improved performance of the proposed CNN method. Optimization of hyperparameters significantly impacted the ability of the proposed model to converge to a good solution and generalize well to unseen data.

The correlation between the observed data and the estimated data values based on various models has also been tested in this study using a perfect, dependable line (Y = X). This makes the process of evaluating the effectiveness and dependability of the models practical. Figures 5, 8, and 11 show the expected and observed data in scattered spots for the various provinces. From each model, the correlation coefficient between the estimated and observed data values is rectified. The correlation coefficient, which goes from −1 to 1, quantifies the linear link between two variables. An inverse relationship is shown by a negative correlation, whereas a direct relationship is indicated by a positive correlation. The strength of the linear link increases with the correlation coefficient’s proximity to 1 (or −1). To produce correlation coefficients for every model-observed data pair, this method is done for every model. The calculated and observed data values thus show a strong linear relationship when the correlation coefficient is around 1 (or −1). Better agreement between the models and the observations is shown by higher correlation coefficients. These procedures evaluate the correlation and agreement between the estimated data values from various models and the observed data. The findings in the data show that the suggested CNN approach worked as well as it could.

As a result, the fuel reforming estimates are performed by applying the SVM, ANN, LSTM, Bi-LSTM, LSTM-GRU, standard CNN methods existing in the literature to the original dataset created, as well as proposing a different and original CNN architecture from the literature. All layers and sequences of the proposed CNN architecture are original (see Fig. 1). In addition, the parameter values used were obtained by optimizing. In order to do this optimization, the Keras Tuner was embedded in the programming. In the proposed CNN model, both convolutional layers and neuron numbers are tested for optimal conditions. All this shows the novelty aspects of our work.

We have determined that the results are highly promising and yield minimal errors, owing to the utilization of a CNN architecture within our proposed technique, alongside optimal scheduling facilitated by modern AI techniques. This method holds potential to be advantageous for the research community in evaluating performance and determining the appropriate sizing for fuel-reformed coupled FCSs.

### The advantages and limitation of the study

The proposed CNN model offers several practical advantages. Firstly, its versatile application is evident as FCSs find use across diverse sectors like transportation, stationary power generation, and telecommunications, underlining the relevance and adaptability of the CNN model to real-world scenarios. Secondly, the model demonstrates enhanced accuracy in predicting crucial output parameters, such as hydrogen yield and carbon monoxide levels, across various reforming processes involving methane, methanol, and diesel fuels. This heightened precision is essential for optimizing FCS efficiency and overall performance. Moreover, the CNN model’s innovative design, integrating both 3D-CNN and 2D-CNN architectures, facilitates comprehensive feature extraction, enabling thorough analysis of reforming processes and precise estimation of output parameters. Lastly, the employment of the Keras Tuner approach allows for the identification of optimal hyperparameter values, thereby enhancing the model’s efficiency and effectiveness in predicting reforming outcomes, ultimately leading to improved performance and reliability.

The proposed CNN model presents several research limitations that warrant consideration. Firstly, while demonstrating superior performance in predicting reforming outcomes for methane, methanol, and diesel, its generalizability to other fuel types or reforming processes may necessitate further validation and testing to ensure robustness across diverse scenarios. Secondly, the efficacy of the CNN model is heavily reliant on the availability and quality of input data; insufficient or noisy data may compromise the model’s accuracy and reliability, emphasizing the need for meticulous data collection and preprocessing efforts. Additionally, the computational demands associated with training and optimizing the CNN model, particularly when employing both 3D-CNN and 2D-CNN architectures, may pose challenges for researchers with limited computational resources or infrastructure. Lastly, while the CNN model yields accurate predictions, the intricate nature of deep learning architectures may hinder interpretability, complicating the understanding of underlying mechanisms driving the model’s predictions and necessitating further exploration in this aspect.

## Conclusions

Hydrogen yield and carbon monoxide vol.% in the reformate syngas are critical parameters for fuel cell powered systems. In addition, methane conversion should be optimized to increase hydrogen production in the methane reforming process. In the present work, a deep CNN strategy is proposed to predict important output parameters of methane, methanol, and diesel reforming processes. For forecasting these parameters, the proposed CNN model is often undertrained. Python programming and libraries are used to model and code the proposed algorithm. In terms of RMSE, MAE, MAPE, and R^2^ metrics that measure prediction performance, the proposed approach has been compared to the SVM, ANN, LSTM, LSTM-GRU, Bi-LSTM, and standard CNN. The results show that the proposed method has higher accuracy than other AI methods. In comparison to alternative methods, the proposed approach demonstrates superior accuracy in estimating carbon monoxide vol.% and hydrogen yield in syngas resulting from methane reforming. Additionally, the proposed method exhibits similarly precise estimations for the reforming processes of methanol and diesel. Notably, when compared to the standard CNN, the proposed CNN model showcases significantly improved performance metrics for carbon monoxide prediction. We concluded that the findings are quite successful and provide the least error since we created a CNN architecture in the proposed technique with optimal scheduling using modern AI methods. The proposed method can be beneficial to the research community for performance estimation and sizing of fuel reformed coupled FCSs. Furthermore, the dataset can be updated for future studies to implement the proposed deep and ML methods in this study.

It has been intended to forecast the reforming of methane, methanol, and diesel using more adaptive deep neural networks in further investigations. It should be noted that further studies can be conducted on different problems to understand limitations and advantageous of the proposed AI technique in this study.

## Supplemental Information

10.7717/peerj-cs.2113/supp-1 Supplemental Information 1 Dataset and Source Code.

## Nomenclature

AI: Artificial intelligence
ANN: Artificial neural network
BRM: Bireforming of methane
CNN: Convolutional neural network
DT: Decision tree
GPR: Gaussian process regression
HT-PEMFC: High-temperature proton-exchange membrane fuel cell
LR: Linear regression
LSTM: Long short term memory
LSTM-GRU: Long short term memory gate recurrent unit
MAE: Mean absolute error
MAPE: Mean absolute percentage error
ML: Machine learning
MSE: Mean square error
PEM: Proton-exchange membrane
PSO: Particle swarm optimization
R: Regression
RAM: Random access memory
R^2^: Coefficient of determination
ReLU: Rectified linear unit
RF: Random forest
RMSE: Root mean square error
SMR: Steam methanol reforming
SOFC: Solid oxide fuel cell
SVM: Support vector machine
SVR: Support vector regression
1D: One-Dimensional
2D: Two-Dimensional
3D: Three-Dimensional

## Additional Information and Declarations

### Data Availability

The following information was supplied regarding data availability: The code and data are available in the Supplemental File.
